# 1482. Combined Test and Treat Campaigns for HIV, Hepatitis B and Hepatitis C: A Systematic Review to Provide Evidence to Support WHO Treatment Guidelines

**DOI:** 10.1093/ofid/ofad500.1318

**Published:** 2023-11-27

**Authors:** Natasha Beard, Andrew Hill

**Affiliations:** Imperial College London, Tunbridge Wells, England, United Kingdom; University of Liverpool, London, England, United Kingdom

## Abstract

**Background:**

Over 38 million individuals are living with HIV, 296 million with chronic Hepatitis B (HBV) and 58 million with chronic Hepatitis C (HCV). Globally, despite successful and cost-effective treatments for these blood-borne viruses (BBVs), over 1.7 million people die per annum. HIV testing is common practise in many countries, yet HBV and HCV screening is often neglected. This systematic review aims to identify the prevalence of the aforementioned BBVs and discuss the cost-effectiveness of implementing combined testing and treatment for HIV, HBV and HCV as one package.

**Methods:**

MEDLINE, Embase and Global Health were searched to identify papers published between 1st January 2013 and 24th February 2023. Included studies reported the prevalence of HIV (anti-HIV 1/2 antibodies), HBV (Hepatitis B surface antigen) and HCV (anti-HCV antibody). Results were stratified into risk groups: blood donors, general population, healthcare attendees, homeless individuals, men who have sex with men (MSM), people who use drugs (PWUD), pregnant women, prisoners, and refugees.

**Results:**

175 studies conducted in 56 countries sampling over 14 million individuals met the eligibility criteria and were included. The mean prevalence of HIV, HBV and HCV was 3.12% (± 7.71%), 4.01% (±5.80%) and 6.70% (±14.64%) respectively. The mean number of individuals testing positive for at least one BBV was 11.83% (±16.86%); with the greatest burden of disease being reported in MSM and PWUD. Therefore, if combined testing was implemented on a global scale, for every 3 individuals diagnosed with HIV, another 4 would be diagnosed with HBV and 7 with HCV.

**Table 1:**
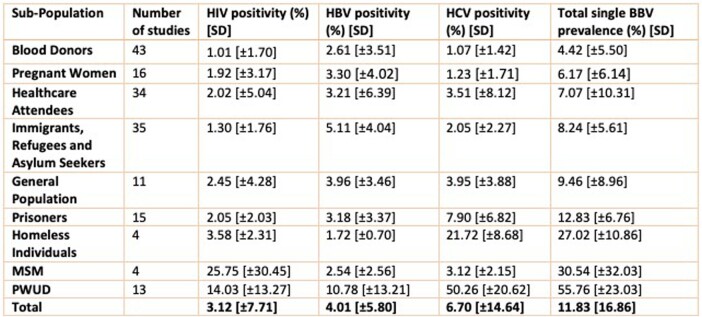
Prevalence of HIV, HBV and HCV and the total testing positive for at least one blood-borne virus in included risk groups

**Figure 1:**
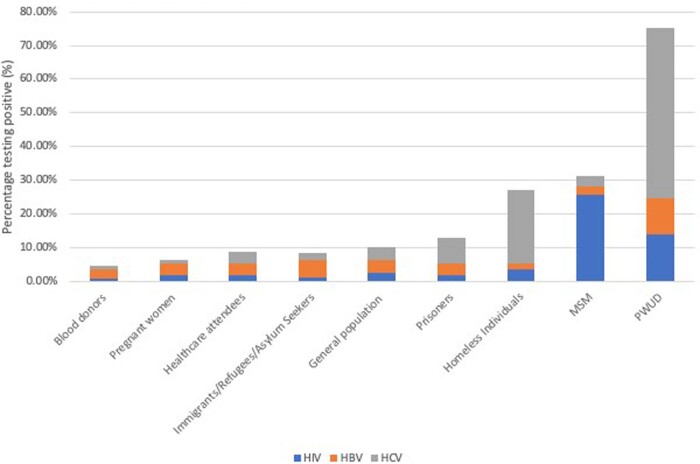
A Bar Chart demonstrating the prevalence of HIV, HBV and HCV in each included risk group

**Conclusion:**

The cost of manufacturing a triple BBV test is $1. Additionally, HIV can be treated for $60/year using TDF/FTC+DTG, HBV for $32/year using generic TDF and HCV can be cured for $79 via generic Sof/Vel, thus providing a cost-effective package solution that could be implemented in the majority of countries. The results of this paper highlight a potential avenue for healthcare improvement by expanding common place isolated HIV testing to combination test and treat programmes. It is only through global diagnosis of all three BBVs, coupled with successful linkage to care, that the Sustainable Development Goal of elimination of these BBV epidemics by 2030 will be achieved.

**Disclosures:**

**All Authors**: No reported disclosures

